# Hesperidin Inhibits Oral Cancer Cell Growth via Apoptosis and Inflammatory Signaling-Mediated Mechanisms: Evidence From In Vitro and In Silico Analyses

**DOI:** 10.7759/cureus.53458

**Published:** 2024-02-02

**Authors:** Selvaraj Jayaraman, Sathanraj Natararaj, Vishnu Priya Veeraraghavan

**Affiliations:** 1 Department of Biochemistry, Centre of Molecular Medicine and Diagnostics (COMManD) Saveetha Dental College and Hospitals, Saveetha Institute of Medical and Technical Sciences, Saveetha University, Chennai, IND

**Keywords:** health and well-being, apoptosis molecular targets, pro-inflammatory signalling, kb cells, oral carcinoma, hesperidin

## Abstract

Background

Oral carcinoma presents a significant health challenge, prompting the need for innovative therapeutic approaches. Elevation of inflammatory mediators, including tumor necrosis factor-alpha (TNF-α) and interleukin-6 (IL-6), has promoted cellular proliferation, inhibited apoptosis, and fostered oral cancer progression through complex signaling pathways. Hesperidin, a flavanone glycoside found in citrus fruits, is of keen interest in this study as it has been proven to have multiple health benefits through in vivo and in vitro studies. However, the mechanism behind the anticancer activity of hesperidin in oral carcinoma remains obscure.

Aim

The study aimed to explore the anticancer potential of hesperidin on human oral cancer cells (KB cells) by modulating pro-inflammatory and apoptotic signaling mechanisms.

Methods

Cancer cell growth inhibitory activity was assessed using the MTT (3-(4,5-Dimethylthiazol-2-yl)-2,5-Diphenyltetrazolium Bromide) assay. Gene expression analysis was performed using real-time RT-PCR analysis. In addition, in silico docking analysis was conducted to confirm the binding affinity of hesperidin with pro-inflammatory and apoptosis signaling molecules. The data were analyzed using one-way ANOVA and the "t" test.

Results

Utilizing the MTT assay, a dose-dependent cytotoxic effect of hesperidin was unveiled, with a remarkable IC50 value indicative of its potent inhibition of cell proliferation. Complementing these findings (p<0.05), qRT-PCR analysis demonstrated hesperidin's regulatory influence on key molecular targets within the KB cell line. Hesperidin treatment resulted in a noteworthy reduction in TNF-α, interleukin-1 beta (IL-1-β), IL-6, nuclear factor kappa-light-chain-enhancer of activated B cells (NF-κB), and B-cell lymphoma 2 (Bcl-2) mRNA expression levels (p<0.05), highlighting its inhibitory role in cell proliferation, migration, and inflammation processes. Simultaneously, hesperidin promoted the expression of BAX mRNA (p<0.05), indicating an enhancement in cell death. Molecular docking simulations further revealed robust binding affinities between hesperidin and target proteins, suggesting its potential to disrupt cellular functions and inflammatory signaling pathways in oral cancer cells.

Conclusion

The cytotoxic effects on the KB cell line and its anti-inflammatory properties position hesperidin as a compelling candidate for further exploration in the quest for effective oral carcinoma treatments. These findings shed light on the intricate molecular mechanisms underlying hesperidin's promise as a therapeutic agent against oral carcinoma.

## Introduction

Oral cancer, referring to malignant neoplasms originating in the oral cavity, including areas such as the tongue, lips, and throat, is a global health concern [1, 2]. Oral carcinoma is characterized by rapid proliferation, differentiation, and the potential to metastasize from the primary site to various regions of the body system [3]. Numerous treatment modalities have been explored for treating oral cancers [4]. Epidemiological data reveal a concerning rise in both the frequency (300,000 cases) and death (145,000 cases) associated with oral cancer [5]. GLOBOCAN (Global Cancer Observatory) reports highlight that oral cancer incidence rates and age-standardized incidence rates are most pronounced in wealthier nations, whereas mortality rates are more prevalent in underdeveloped countries, emphasizing disparities in oral cancer outcomes rooted in social inequalities [6]. Furthermore, oral cancers represent a significant global health burden, affecting individuals from diverse backgrounds. The comprehension of their pathogenesis and the development of effective treatments continue to pose ongoing challenges [7].

One important pathological factor associated with oral cancer is prolonged inflammation. Chronic inflammatory conditions such as oral lichen planus and leukoplakia are strongly associated with the development of cancer in the oral cavity [8]. Inflammatory responses triggered by various factors, including nicotine and fermented beverage use, chronic viral infections, and mechanical irritation, can contribute to the chronic inflammatory environment within the oral cavity. Proinflammatory cytokines and chemokines play a pivotal role in the progression of oral carcinoma [9]. These include tumor necrosis factor-alpha (TNF-α), interleukin-6 (IL-6), IL-1, and various chemokines. These molecules can stimulate inflammation, promote cell division, and facilitate angiogenesis, which are regarded as hallmarks of cancer development. The nuclear factor-kappa B (NF-κB) signaling pathway is a key regulator of proinflammatory responses. Activation of NF-κB promotes the expression of proinflammatory genes. Aberrant activation of NF-κB is commonly observed in oral cancer, contributing to tumor growth, invasion, and resistance to cell death [10].

There has been a notable recent trend towards exploring potential plant-based compounds as novel drugs in oral cancer therapy. Medicinal plants are often considered to possess lower toxicity levels and fewer side effects, or no side effects, compared to chemically synthesized drugs [11]. Hence, the search for more potent anticancer drugs, preferably derived from cost-effective dietary sources with minimal or no side effects, is imperative.

Recently, there has been significant interest in the potential benefits of citrus fruits and their components, particularly flavonoids. This heightened interest stems from their association with a lowered risk of specific chronic illnesses and enhanced longevity [12]. Hesperidin, a flavonoid compound found in citrus fruits, has been extensively studied for its pharmacological effects to identify its potential health benefits. This research aims to uncover the advantageous properties of hesperidin within the context of citrus, a globally significant fruit known for its nutritional richness and abundance of bioactive compounds [13]. It showcases hepatoprotective, antiviral, anti-inflammatory, antihyperlipidemic, antihyperglycemic, antitumor, antioxidant, antifungal, and immune regulatory activity produced by CCL4 [14, 15], and neuroprotective, antidepressant, and cardioprotective effects in diabetic rats [16,17,18].

It has also been reported that hesperidin may play a chemopreventive role, potentially helping to prevent the initiation of cancer or the development of precancerous cells. Hesperidin has been shown to modulate the immune response, thereby improving the host’s ability to identify and eliminate cancerous cells [19]. However, the mechanisms by which hesperidin can inhibit the growth of oral carcinogenesis by modulating pro-inflammatory and apoptotic signaling molecules, which play a central role in oral cancer, remain unknown. Hence, this study attempted to discover the anticancer activity of hesperidin in oral cancer cells.

## Materials and methods

Culture of cells

Human oral cancer (KB cell line) cells were purchased from the National Centre for Cell Science (NCCS) and cultured at ambient temperature according to protocols.

Cell viability by MTT ((3-(4,5-Dimethylthiazol-2-yl)-2,5-Diphenyltetrazolium Bromide)) assay

After the KB cells reached confluence, 6000 cells were seeded in a plate, incubated, and then treated with hesperidin at various time intervals and doses. Following 48 hours of incubation, 50 μL of MTT reagent from Abcam was added and incubated in the dark for 3-4 hours. Subsequently, the medium was gently aspirated, and 100μL of dimethyl sulfoxide (DMSO) solvent was introduced into all the wells. The mixture was covered with foil, agitated for 5 minutes, and measured at 590 nm.

mRNA expression by RT-PCR

To analyze the gene expression pattern of molecules in response to hesperidin treatment, 5 × 10^6 cells were loaded into separate wells of a 6-well plate, and hesperidin was added with a serum-free medium at a 24-hour incubation. Using the TRIR kit, we extracted the total RNA from the cells. The RNA was then subjected to complementary DNA synthesis using a reverse transcriptase kit. The cDNA was utilized for mRNA studies using real-time PCR.

Molecular docking analysis

The study delved into the investigation of binding interactions between hesperidin and crucial apoptosis-regulating proteins, specifically for pro-inflammatory and apoptotic proteins, and a total of 100 genetic algorithm runs were conducted to explore various binding conformations and orientations, comprehensively examining the potential binding interactions between hesperidin and these apoptosis-regulating proteins.

Statistical analysis

The data were presented as mean ± SD. The statistical significance of the findings was assessed through statistical analysis performed with GraphPad Prism 8 software. A t-test was applied to assess the significance of the data, with the representation of p-value as ***< 0.001, **< 0.01, and *< 0.05.

## Results

Effect of hesperidin on the inhibition of oral cancer cell growth

**Figure 1:**
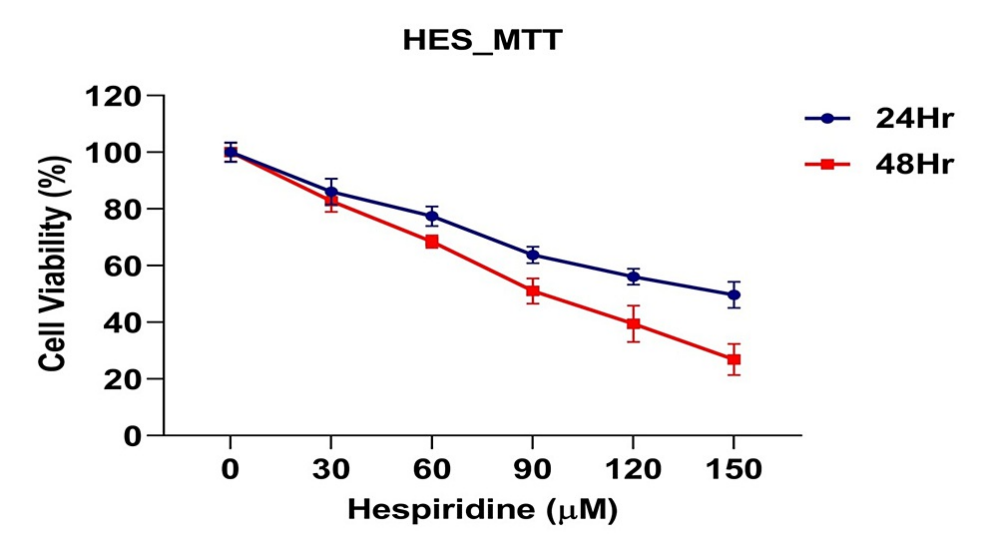
Effect of hesperidin on the growth of oral cancer cells using the MTT assay method. Inhibition of oral cancer cell growth in human KB cells by hesperidin. Cells were cultured and treated with varying concentrations of hesperidin (0, 30, 60, 90, 120, and 150 µM) for 24 and 48 hours. Cell viability was assessed, and the viability of KB cells was expressed as a percentage. Each line represents the mean ± SD of three independent observations.

The impact of hesperidin on the cytotoxicity of the KB cell line was evaluated through an MTT assay. In the MTT assay, hesperidin demonstrated a dose-response cytotoxic effect, with an IC50 value of 89.17μM, highlighting its efficacy in inhibiting cell proliferation over 48-hour intervals (Figure 1). These findings align with observations from the MTT assay, collectively providing strong evidence of hesperidin's cytotoxic effects on the KB cell line.

Effects of hesperidin on proinflammatory signaling target molecules in KB cells

**Figure 2:**
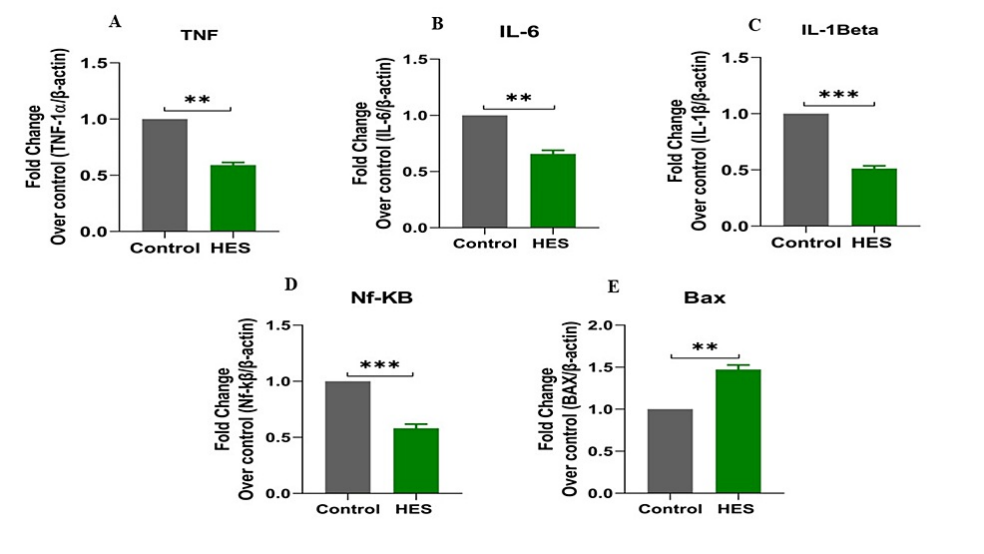
Effects of hesperidin on gene expression of Inflammatory targets (TNF-1α, IL-1β, IL-6, Nf-KB, and BAX) in KB cells. The Cells were cultured and treated with or without hesperidin (0, 30, 60, 90, 120 and 150µM) with at 24 and 48 hrs incubation. mRNA expression of genes was analysed by Real‐Time PCR using SYBR green dye. Target gene expression was normalized with β‐actin mRNA and the results were expressed as fold change over untreated control group. In the context of t-tests, the significance was at the levels of p<0.05 (*p<0.05,**p < 0.01, ***p<0.001, ****p<0.001). “*” denotes statistical significance between control and hesperidin treated groups.

The qRT-PCR analysis unveiled the regulatory impact of hesperidin on critical molecular targets within the KB cell line. Upon treatment with hesperidin, a noteworthy decrease in NF-κB mRNA, IL-6 mRNA, IL-1β mRNA, and TNF-α mRNA expression levels was observed. This decrease signifies the inhibitory effects of hesperidin on these pivotal proteins associated with processes like cell proliferation, migration, and inflammatory activity. Simultaneously, hesperidin therapy resulted in a substantial elevation in BAX mRNA expression, which suggests an enhancement in cell death, as shown in Figure 2. These findings collectively indicate that hesperidin exerts its inhibitory influence on the KB cell line by suppressing TNF-α, IL-1β, IL-6, and NF-κB while concurrently promoting the expression of BAX. This data provides valuable insights into the molecular mechanisms underlying its potential as a promising therapeutic agent against oral carcinoma.

Molecular docking studies

**Figure 3:**
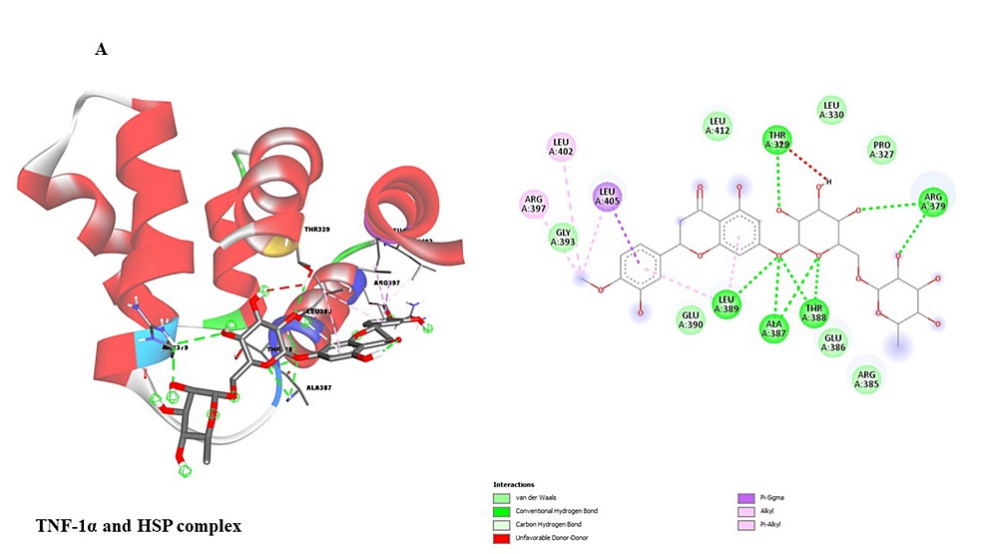
Showing the molecular docking analysis of hesperidin with TNF-α protein. TNF-α: Tumor necrosis factor-alpha.

**Figure 4:**
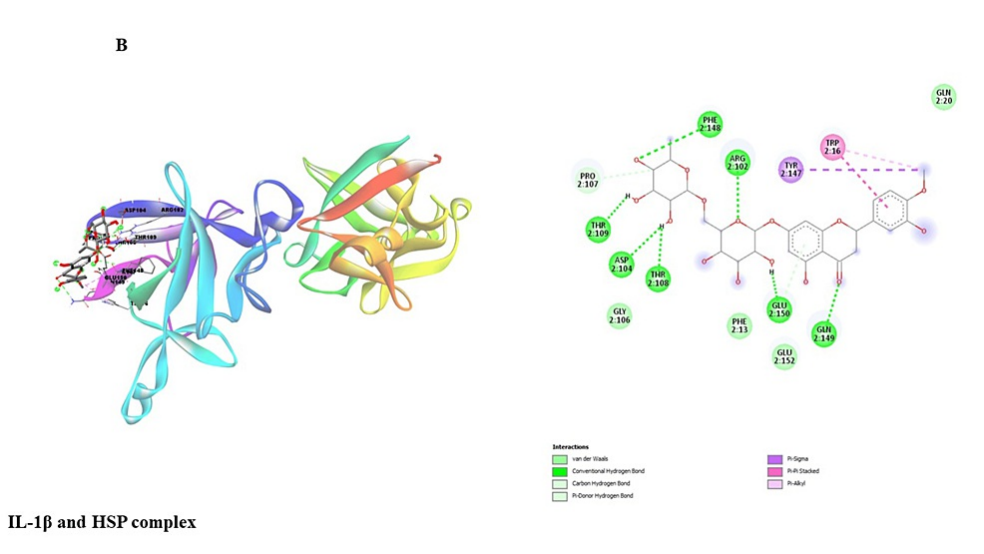
Showing the molecular interaction of hesperidin with IL-1β protein. IL-1β: Interleukin-1 beta.

**Figure 5:**
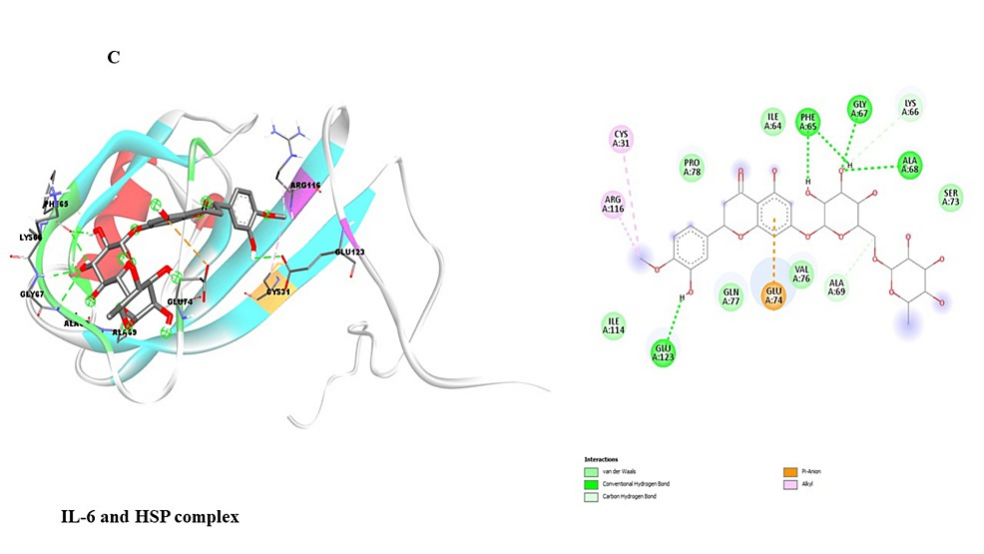
Represents the binding interaction of hesperidin with the IL-6 protein complex. IL-6: Interleukin-6.

**Figure 6:**
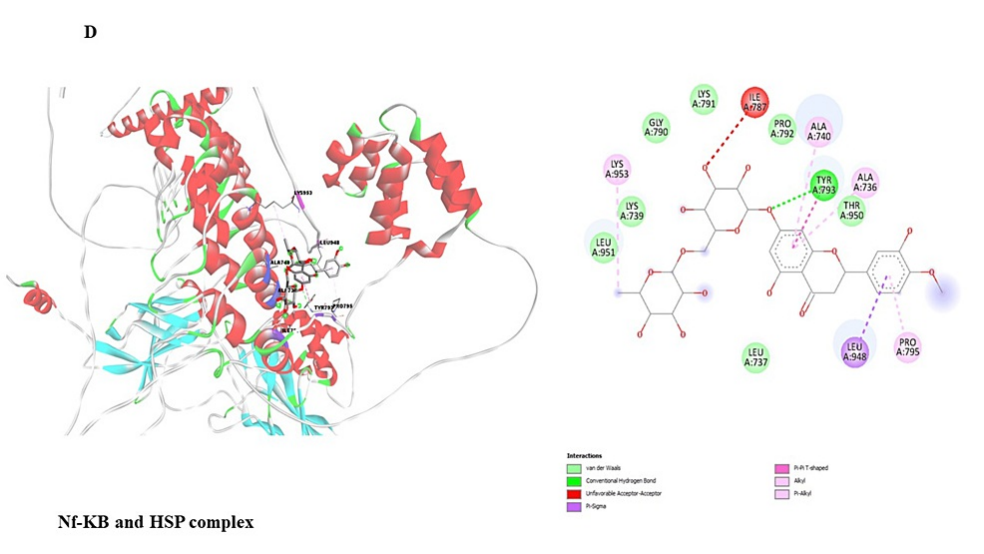
Represents the binding interaction of hesperidin against NF-kB protein complex.

**Figure 7:**
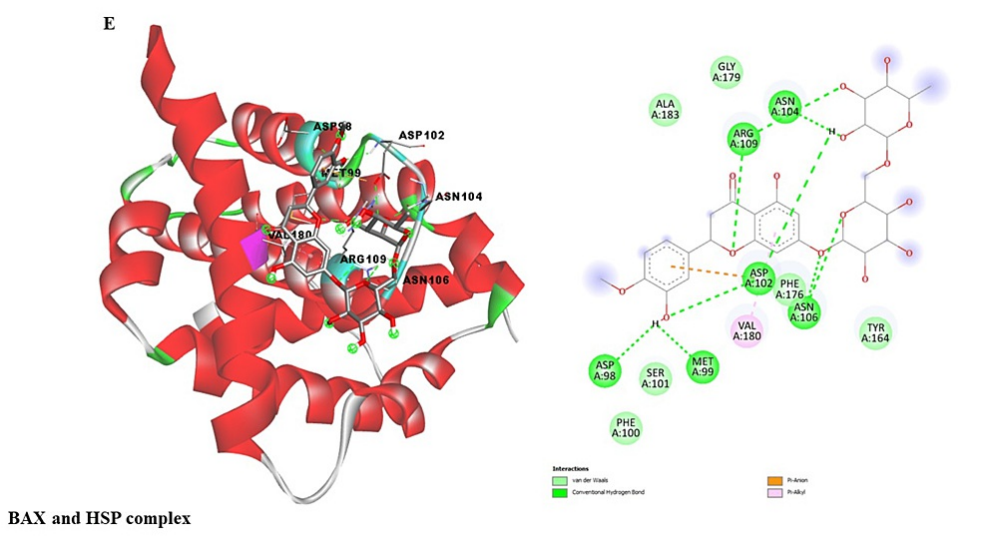
Represents the binding interaction of hesperidin against BAX protein complex.

This study investigated potential binding interactions between hesperidin and critical proteins involved in inflammatory regulation (Table 1). Figures 3-7 depict the 3D structures of hesperidin docked with BAX, NF-κB, TNF-α, IL-6, and IL-1β. Table 2 summarizes critical parameters, including binding energy, the number of hydrogen bonds, and the residues engaged in hydrogen bond formation for each drug-protein interaction. The outcomes of the docking simulations demonstrated strong binding affinities between hesperidin and the proteins it targets. These findings imply that hesperidin could substantially interact with these proteins, potentially disrupting their cellular functions and inflammatory signaling pathways.

**Table 1:** Molecular docking results showing the binding interaction of hesperidin with proinflammatory and apoptotic signaling molecules. TNF-α: Tumor necrosis factor-alpha; IL-1β: Interleukin-1 beta; IL-6: Interleukin-6.

Drug name	Targets	Binding affinity (Kcal/mol)	No. of H bonds	Amino acid residues
Hesperidin	TNF-1α	-7.7	4	LEU389, ALA387, THR388,THR329, ARG379
	IL-1β	-7.6	3	THR109, ASP104, THR108, GLU150
	IL-6	-7.3	3	GLU123, PHE65, GLY67, ALA68
	Nf-KB	-8.3	1	TYR793
	BAX	-7.7	2	ASP98, ARG109

**Table 2:** List of gene-specific primers used. F: Forward; R: Reverse; TNF-α: Tumor necrosis factor-alpha; IL-1β: Interleukin-1 beta; IL-6: Interleukin-6.

Gene(s)	Primer 5’-3’
TNF-1α	F- ACAAGCCTGTAGCCCATGTT R- AAAGTAGACCTGCCCAGACT
Bax	F-TTCTGACGGCAACTTCAACTG R-TGAGGAGTCTCACCCAACCA
IL-1 β	F- GGATATGGAGCAACAAGTGG R- ATGTACCAGTTGGGGAACTG
IL-6	F-TCAATGAGGAGACTTGCCTG R- GATGAGTTGTCATGTCCTGC
Nf-Kβ	F- ATGCATGCTAGCTAGCTAGC R- GCTAGCTAGCTAGCATGCAT
β-actin	F-AACAAGATGAGATTGGCA R-AGTGGGGTGGCTTTTAGGAT

## Discussion

This encompassing term includes various anatomical structures such as the lips, tongue, gums, the inner lining of the cheeks, the roof and floor of the mouth, and even the tonsils [20]. This type of cancer falls under the broader category of head and neck cancer, with a predominant form being oral carcinoma. It arises from the squamous cells that form the lining of the oral cavity [21]. Traditionally, the standard treatments for oral cancer involve a combination of surgical interventions, radiation therapy, and chemotherapy [22]. These treatments aim to kill the cancerous cells, aiming to cure or manage the disease. However, alongside these conventional therapies, there is a growing interest in exploring the potential therapeutic advantages of natural compounds and medications derived from plants [23]. This emerging field of research seeks to harness the inherent characteristics of several natural compounds for managing oral cancer. These compounds, often obtained from plant sources, are investigated for their ability to hinder the development and spread of oncogenic cells, initiate apoptotic pathways in abnormal cells, and mitigate the side effects of conventional cancer treatments [24-26].

This study comprehensively evaluates the potential therapeutic efficacy of hesperidin against oral carcinoma, focusing on its cytotoxic effects on the KB cell line, its regulatory impact on vital molecular targets, and its binding interactions with proteins involved in inflammatory regulation. The MTT assay results demonstrate that hesperidin exerts a dose-dependent cytotoxic effect on the KB cell line, with a highlighted IC50 value indicating its potency in inhibiting cell proliferation. As illustrated in Figure 1, this dose-response relationship reinforces the evidence of hesperidin's cytotoxicity in KB cells. These findings are particularly promising as they suggest that hesperidin could be a valuable component in therapies to reduce the growth of oral carcinoma cells.

The qRT-PCR analysis further elucidates the underlying molecular mechanisms of hesperidin's effects on KB cells. Treatment with hesperidin observed a significant downregulation of mRNA expressions of pro-inflammatory markers (NF-κB, IL-1β, TNF-α, and IL-6), the critical proteins associated with processes such as cell proliferation, migration, and inflammation. Concurrently, hesperidin treatment led to a substantial increase in BAX mRNA expression, indicating an enhancement in cell death and a favorable outcome in combating cancer (Figure 2). These results collectively suggest that hesperidin exerts its inhibitory influence on the KB cell line by suppressing pro-inflammatory and pro-proliferative factors while promoting apoptotic pathways. The subsequent molecular docking study (Figure 3 and Table 2) provides insights into the molecular relationship between hesperidin and essential biomarkers in inflammatory regulation. The robust binding affinities noted for hesperidin and these target proteins imply that hesperidin may effectively interfere with their cellular functions and disrupt inflammatory signaling pathways. This suggests a multi-faceted mechanism of action for hesperidin, involving both direct cytotoxicity and indirect modulation of critical inflammatory targets.

Limitations of the study

The current study offers in vitro experimental studies on a human cell line model evidence of the therapeutic impact of hesperidin on inhibiting oral cancer cells by inhibiting pro-inflammatory signaling molecules and activating BAX (pro-apoptotic protein) signaling. However, the in vivo evaluation of hesperidin on oral cancer-induced models has not been studied, which could bring more in vivo evidence in order to develop hesperidin as a novel therapeutic drug in the treatment of oral cancer.

## Conclusions

The findings of this study establish hesperidin as a compelling candidate for further exploration and development in the context of oral carcinoma therapy. Its demonstrated cytotoxicity, regulatory effects on crucial molecular targets, and robust binding interactions with inflammatory proteins collectively provide a robust foundation for future research and clinical investigations. In conclusion, hesperidin holds promise as a valuable component in the ongoing pursuit of improved therapeutic strategies for treating oral carcinoma, instilling hope for more effective treatments in the foreseeable future.

## References

[1] Radhakrishnan R, Shrestha B, Bajracharya D (2018). Oral cancer - an overview.

[2] Borse V, Konwar AN, Buragohain P (2020). Oral cancer diagnosis and perspectives in India. Sens Int.

[3] Lu C, Lewis JS Jr, Dupont WD, Plummer WD Jr, Janowczyk A, Madabhushi A (2017). An oral cavity squamous cell carcinoma quantitative histomorphometric-based image classifier of nuclear morphology can risk stratify patients for disease-specific survival. Mod Pathol.

[4] Ruddy K, Mayer E, Partridge A (2009). Patient adherence and persistence with oral anticancer treatment. CA Cancer J Clin.

[5] Sung H, Ferlay J, Siegel RL, Laversanne M, Soerjomataram I, Jemal A, Bray F (2021). Global Cancer Statistics 2020: GLOBOCAN estimates of incidence and mortality worldwide for 36 cancers in 185 countries. CA Cancer J Clin.

[6] Maslach C, Leiter MP (2016). Understanding the burnout experience: recent research and its implications for psychiatry. World Psychiatry.

[7] Niklander SE (2021). Inflammatory mediators in oral cancer: pathogenic mechanisms and diagnostic potential. Front Oral Health.

[8] Tezal M (2012). Interaction between Chronic Inflammation and Oral HPV Infection in the Etiology of Head and Neck Cancers. Int J Otolaryngol.

[9] Hirano T (2021). IL-6 in inflammation, autoimmunity and cancer. Int Immunol.

[10] Jamal Gilani S, Nasser Bin-Jumah M, Al-Abbasi FA, Shahid Nadeem M, Afzal M, Sayyed N, Kazmi I (2021). Fustin ameliorates hyperglycemia in streptozotocin induced type-2 diabetes via modulating glutathione/Superoxide dismutase/Catalase expressions, suppress lipid peroxidation and regulates histopathological changes. Saudi J Biol Sci.

[11] Chen H, Ward MH, Graubard BI (2002). Dietary patterns and adenocarcinoma of the esophagus and distal stomach. Am J Clin Nutr.

[12] Malterud KE, Rydland KM (2000). Inhibitors of 15-lipoxygenase from orange peel. J Agric Food Chem.

[13] Chiba H, Uehara M, Wu J (2003). Hesperidin, a citrus flavonoid, inhibits bone loss and decreases serum and hepatic lipids in ovariectomized mice. J Nutr.

[14] Jayaraman R, Subramani S, Sheik Abdullah SH, Udaiyar M (2018). Antihyperglycemic effect of hesperetin, a citrus flavonoid, extenuates hyperglycemia and exploring the potential role in antioxidant and antihyperlipidemic in streptozotocin-induced diabetic rats. Biomed Pharmacother.

[15] Ashafaq M, Varshney L, Khan MH, Salman M, Naseem M, Wajid S, Parvez S (2014). Neuromodulatory effects of hesperidin in mitigating oxidative stress in streptozotocin induced diabetes. Biomed Res Int.

[16] El-Marasy SA, Abdallah HM, El-Shenawy SM, El-Khatib AS, El-Shabrawy OA, Kenawy SA (2014). Anti-depressant effect of hesperidin in diabetic rats. Can J Physiol Pharmacol.

[17] Vabeiryureilai M, Lalrinzuali K, Jagetia GC (2019). Chemopreventive effect of hesperidin, a citrus bioflavonoid in two stage skin carcinogenesis in Swiss albino mice. Heliyon.

[18] Liu H, Huang Y, Huang M (2022). Current status, opportunities, and challenges of exosomes in oral cancer diagnosis and treatment. Int J Nanomedicine.

[19] Pan Z, Dong H, Huang N, Fang J (2022). Oxidative stress and inflammation regulation of sirtuins: new insights into common oral diseases. Front Physiol.

[20] Yasuoka T, Yonemoto K, Kato Y, Tatematsu N (2000). Squamous cell carcinoma arising in a dentigerous cyst. J Oral Maxillofac Surg.

[21] Nguyen NP, Sallah S, Karlsson U, Antoine JE (2002). Combined chemotherapy and radiation therapy for head and neck malignancies: quality of life issues. Cancer.

[22] Coimbra M, Isacchi B, van Bloois L (2011). Improving solubility and chemical stability of natural compounds for medicinal use by incorporation into liposomes. Int J Pharm.

[23] Baudino TA (2015). Targeted Cancer Therapy: The Next Generation of Cancer Treatment. Curr Drug Discov Technol.

[24] Pérez-Herrero E, Fernández-Medarde A (2015). Advanced targeted therapies in cancer: drug nanocarriers, the future of chemotherapy. Eur J Pharm Biopharm.

[25] Fang B (2014). Development of synthetic lethality anticancer therapeutics. J Med Chem.

[26] Jayaraman S, Natarajan SR, Veeraraghavan VP, Jasmine S (2023). Unveiling the anti-cancer mechanisms of calotropin: Insights into cell growth inhibition, cell cycle arrest, and metabolic regulation in human oral squamous carcinoma cells (HSC-3). J Oral Biol Craniofac Res.

